# Case Report: Coexistence of Multiple Myeloma and Auricular Chondritis in VEXAS Syndrome

**DOI:** 10.3389/fimmu.2022.897722

**Published:** 2022-06-09

**Authors:** Haruki Matsumoto, Yuya Fujita, Masahiko Fukatsu, Takayuki Ikezoe, Kohei Yokose, Tomoyuki Asano, Naomi Tsuchida, Ayaka Maeda, Shuhei Yoshida, Honami Hashimoto, Jumpei Temmoku, Naoki Matsuoka, Makiko Yashiro-Furuya, Shuzo Sato, Mai Murakami, Hidenori Sato, Chiharu Sakuma, Kazumasa Kawashima, Norshalena Shakespear, Yuri Uchiyama, Hiroshi Watanabe, Yohei Kirino, Naomichi Matsumoto, Kiyoshi Migita

**Affiliations:** ^1^Department of Rheumatology, Fukushima Medical University School of Medicine, Fukushima, Japan; ^2^Department of Hematology, Fukushima Medical University School of Medicine, Fukushima, Japan; ^3^Department of Stem Cell and Immune Regulation, Yokohama City University Graduate School of Medicine, Yokohama, Japan; ^4^Department of Human Genetics, Yokohama City University Graduate School of Medicine, Yokohama, Japan; ^5^Department of Rare Disease Genomics, Yokohama City University Hospital, Yokohama, Japan; ^6^Department of Otolaryngology, Fukushima Medical University School of Medicine, Fukushima, Japan; ^7^Department of Gastroenterology, Fukushima Medical University School of Medicine, Fukushima, Japan; ^8^Department of Diagnostic Pathology, Fukushima Medical University School of Medicine, Fukushima, Japan

**Keywords:** auricular chondritis, multiple myeloma, plasma cell dyscrasia, VEXAS syndrome, *UBA1*

## Abstract

Vacuoles, E1 enzyme, X-linked, autoinflammatory, somatic (VEXAS) syndrome is an inflammatory disorder caused by somatic *UBA1* variants, which are sometimes associated with hematological disorders, including myelodysplastic syndrome (MDS). VEXAS syndrome often overlaps with rheumatic diseases, including relapsing polychondritis. Here, we describe a case of VEXAS syndrome with auricular chondritis and exceptional multiple myeloma (MM). An 83-year-old man was diagnosed with MM, which was treated once by lenalidomide hydrate obtaining a partial response, but the patient did not desire further aggressive therapy. Although the treatment was effective, progressive macrocytic anemia and inflammation of both the ears emerged over the following 2 months. The histological examination of the auricle skin revealed that the perichondrial area was infiltrated by inflammatory cells, leading to the diagnosis of auricular chondritis. He was treated with oral prednisolone 40 mg/day, and his symptoms rapidly resolved. The re-evaluation of the histopathological bone marrow findings revealed vacuoles in the myeloid precursor cells without myelodysplasia-related changes. Sanger sequencing of *UBA1* was performed using genomic DNA from peripheral blood leukocytes and revealed a somatic variant (c.122T>C:p.Met41Thr) consistent with VEXAS syndrome. This demonstrates that patients with chondritis can have complications with MM despite the absence of underlying MDS. A strong association exists between *UBA1* variants and the risk of MDS; however, it remains elusive whether somatic *UBA1* variants contribute to the development of plasma cell dyscrasia without MDS. Hence, we discuss the possible relationship between auricular chondritis and MM on a background of VEXAS syndrome.

## Introduction

Vacuoles, E1 enzyme, X-linked, autoinflammatory, somatic (VEXAS) syndrome is a newly identified autoinflammatory disorder characterized by systemic inflammation that affects multiple tissues (1). Because of its wide range of phenotypes, VEXAS syndrome often meets the criteria for rheumatic disease, including that of relapsing polychondritis (RP) (2). In addition to heterogeneous rheumatic manifestations, VEXAS syndrome presents with hematological disorders, including myelodysplastic syndrome (MDS) (3). Patients who possess the myeloid lineage-restricted somatic variants in *UBA1* affecting the Met41 residue of the protein have decreased cellular ubiquitination activity, resulting in hyper-inflammation (4). The majority of patients with VEXAS syndrome are complicated with MDS, but few present with plasma cell dyscrasia such as multiple myeloma (MM) (5). However, the mechanism by which plasma cell neoplasms can be complicated with VEXAS syndrome remains unknown. Here, we identified a somatic *UBA1* variant in a patient with auricular chondritis and MM whose clinical and genetic data will be presented and discussed.

Ethical approval for this study (2019-141) was provided by the Ethics Committee of Fukushima Medical University and the Institutional Review Board of Yokohama City University. Written informed consent was obtained from the present case.

## Case Description

**Figure 1:**
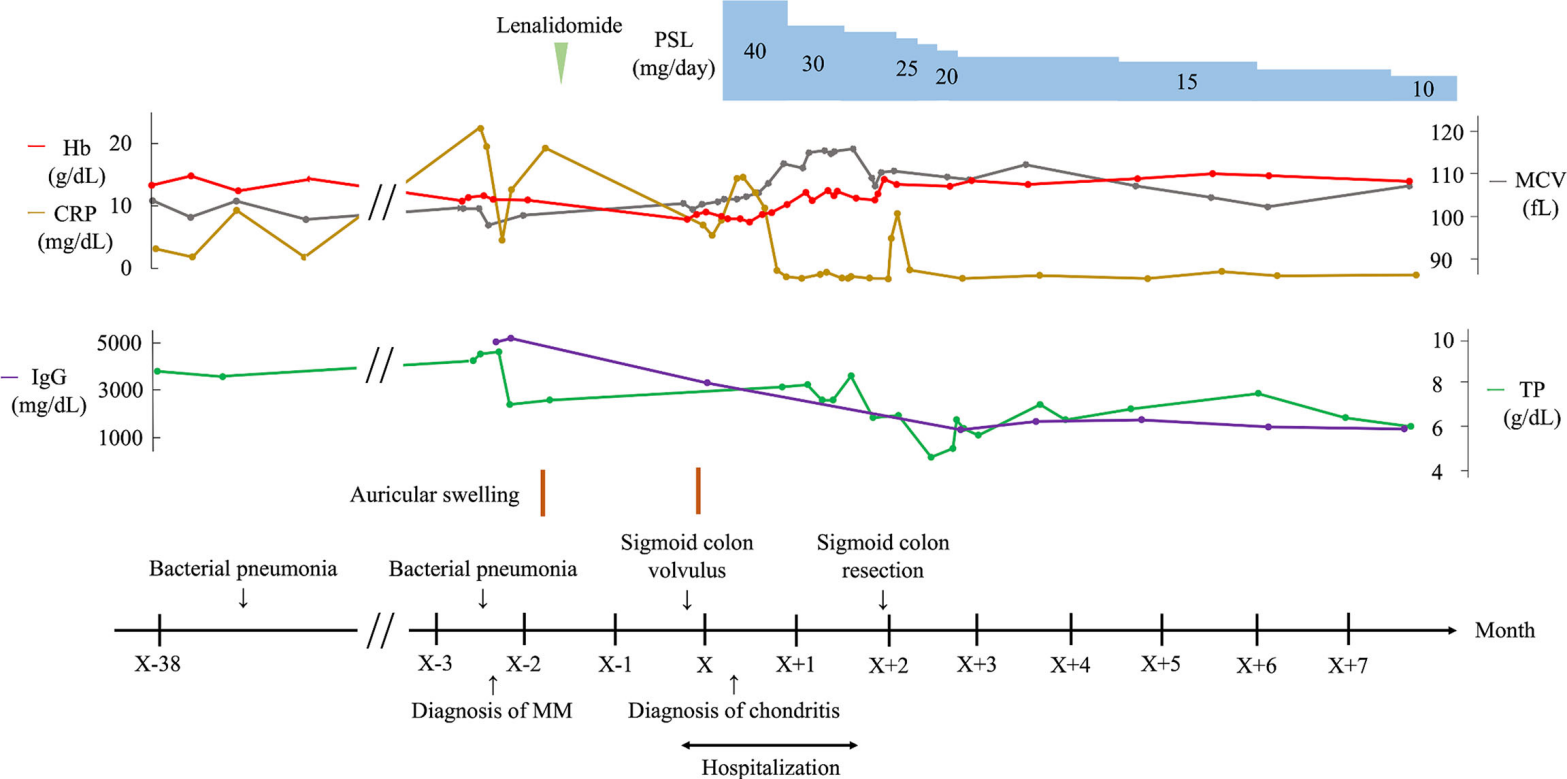
Clinical course of the patient. CRP, C-reactive protein; Hb, hemoglobin; IgG, immunoglobulin G; MCV, mean corpuscular volume; MM, multiple myeloma; PSL, prednisolone; RP, relapsing polychondritis; TP, total protein.

**Figure 2:**
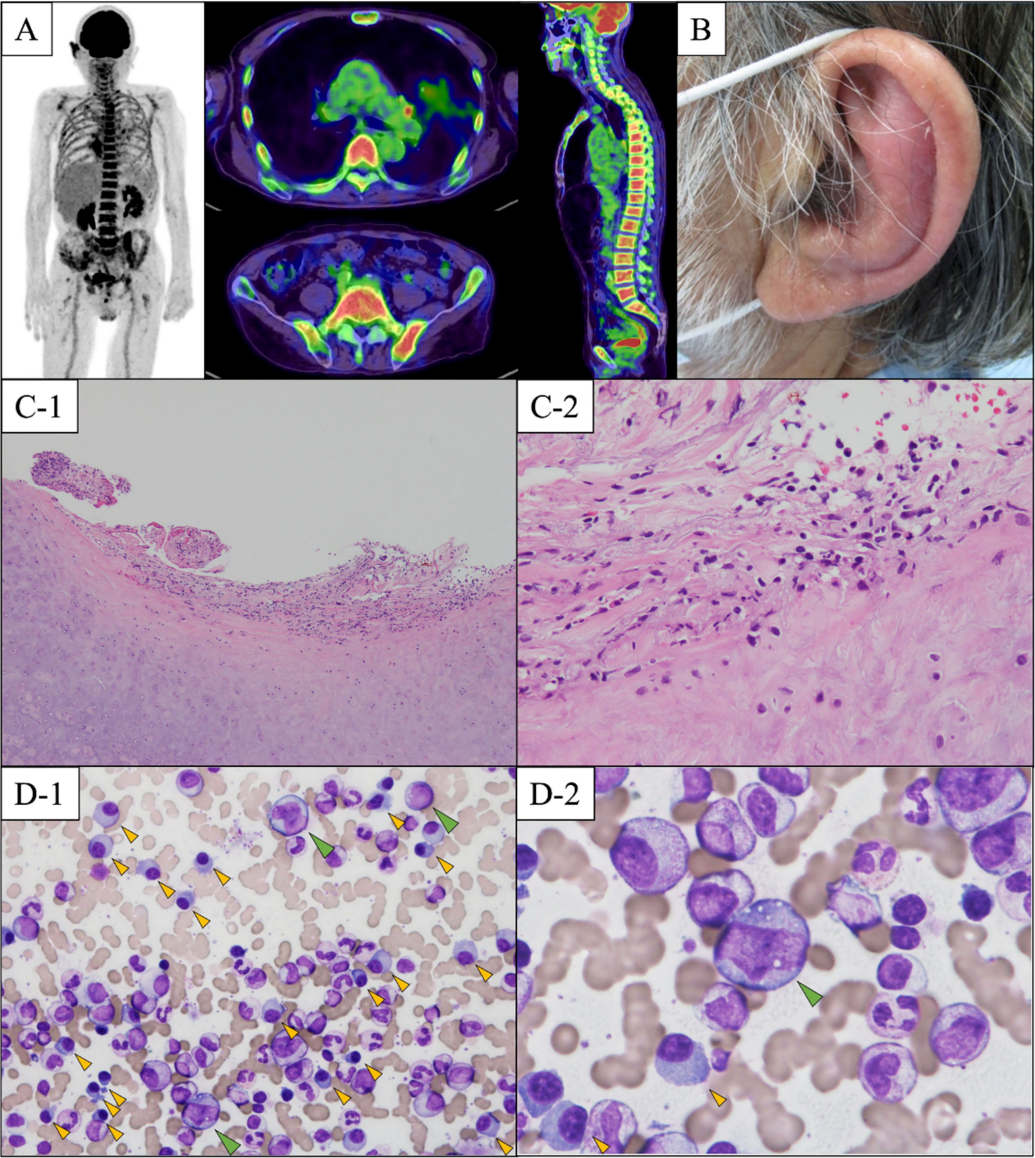
**(A)** Result of ^18^F-fluorodeoxyglucose (FDG) positron emission tomography-computed tomography. There is marked FDG accumulation in the ribs, vertebrae, and ilium. **(B)** A floppy left ear. Our patient had prominent redness and pain in the left auricular region. **(C)** Histological findings of a biopsy specimen from the left auricle. Inflammatory cell infiltration of neutrophils around the auricular cartilage is observed **(C-1)**. In addition, granuloma formation consisting of fibrosis and hypervascularization suggestive of chronic inflammation is observed **(C-2)** (hematoxylin and eosin staining, **(C-1)** original magnification ×40, **(C-2)** original magnification ×100). **(D)** Bone marrow smear findings. **(D-1)** Clonal expansion of plasma cells is shown by the yellow arrows. The bone marrow dysplasia suggesting MDS is not observed. **(D-2)** Vacuolization in granulocytic precursor cells are shown by green arrow. (May–Giemsa staining, **(D-1)** original magnification ×400, **(D-2)** original magnification ×1000).

An 83-year-old Japanese man was admitted to our department for persistent inflammatory reactions. His previous clinical course is shown in **Figure 1**. He had bronchial asthma at the age of 64 years and no abnormal hematological findings till the age of 80 years. Two months before his hospitalization at our department, he was referred to the Department of Hematology for anemia and hypergammaglobulinemia. His laboratory results are shown in **Supplementary Table 1**. Specifically, elevated monoclonal IgG levels at 5,103 mg/dL, anemia (hemoglobin 10.9 g/dL), and hypercalcemia (10.5 mg/dL with albumin correction) were indicative of MM. Bone marrow aspirate showed increased levels of atypical plasma cells at 24.8%. Immunohistochemical analysis using flow cytometry confirmed the presence of clonal plasma cells, which were positive for IgG, κ, cyclin D1, CD38, CD79a, and CD138, and negative for CD19 and CD56; CD19 or CD56 negativity indicated that the expanded plasma cells were myeloma cells (6). However, there were no definitive findings in the myeloid or erythroid cells suggestive of MDS in the BM aspirates and peripheral blood smear. G-banded chromosomal analysis was normal (46, XY). Fluorescence *in situ* hybridization was positive for t(11;14)(q13;q32)/CCND1-IGH, but negative for t(4;14)(p16;q32)/FGFR3-IGH, t(14;16)(q32;q23)/IGH-MAF, and del(17p) TP53. A skeletal survey *via* ^18^F-fluorodeoxyglucose (FDG) positron emission tomography–computed tomography showed diffuse FDG uptake in the ribs, vertebrae, and ilium (**Figure 2A**). He had no other findings related to myeloma-defining events, but had two episodes of bacterial pneumonia. Hence, he was considered to be progressively susceptible to infection and was diagnosed with symptomatic MM stage II based on the revised international staging system (7). He was treated with lenalidomide, but it was discontinued due to accidental sigmoid colon volvulus. The sigmoid colon volvulus was treated endoscopically in our gastroenterology department. He then refused additional treatment for MM; however, he achieved partial response based on the International Myeloma Working Group consensus criteria (8) without further progression.

**Figure 3:**
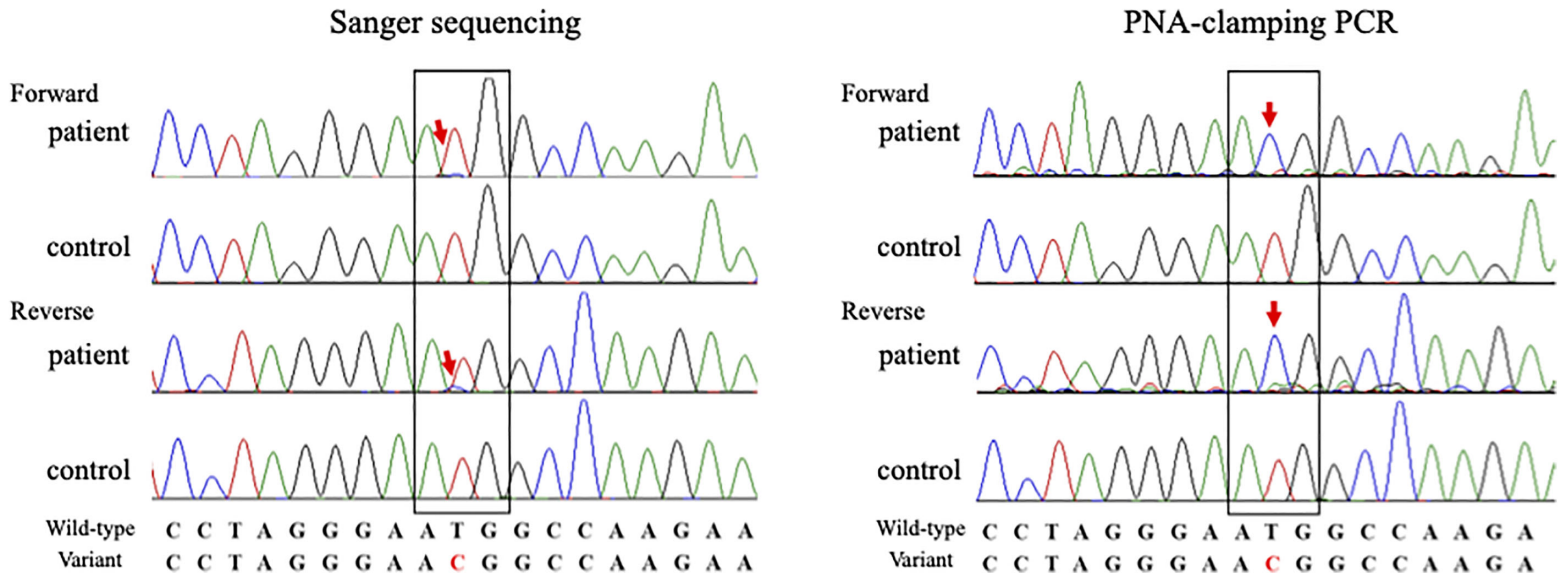
Sanger sequencing (exome sequencing) of genomic DNA from peripheral blood (red arrows) revealed that the patient had the electropherogram’s peak of a somatic variant in *UBA1* (NM_003334.3:c.122T>C:p.Met41Thr). To further validate the low-prevalence somatic variants in *UBA1*, peptide nucleic acid-clamping polymerase chain reaction was performed. The pathogenic variant at c.122T>C:p.Met41Thr is seen (red arrows) in a condition where the wild-type allele was not amplified.

Two months after MM diagnosis, he was referred and admitted to our department for persistent inflammatory reactions. Physical examination revealed a floppy appearance of both the ears (**Figure 2B**), whereas there were no abnormal findings in his nasal and tracheal cartilages, eyes, and skin. Laboratory findings showed anemia, an increased platelet count, a decreased albumin level (1.6 g/dL), and elevated erythrocyte sedimentation rate (100 mm/hr) and CRP level (8.34 mg/dL). IgG levels were also elevated at 3,472 mg/dL. The patient was negative for anticyclic citrullinated peptides antibody, and the antibody to type II collagen was negative (24.0 E·U/mL; normal, <25.0 E·U/mL) (**Supplementary Table 2**). Laboratory changes associated with MM is described in **Supplementary Table 3**. Although the antitype II collagen antibody was negative, auricle erythema suggested the presence of polychondritis. Biopsy was performed on the skin and cartilage of the left auricle, and the histological findings showed cellular infiltrates of lymphocytes, neutrophils, and plasma cells, especially at the cartilage-skin interface, and a reduced number of chondrocytes in areas of cartilage destruction (**Figure 2C**). He did not fulfill McAdam’s criteria for RP (9) and he was diagnosed with auricular chondritis. He received 40 mg of intravenous prednisolone, and inflammation in both the ears improved immediately. Two months following the steroid therapy, the prednisolone (PSL) dose was reduced to 27.5 mg. The inflammation of the auricular region and CRP decreased, and the patient did not show relapse of any symptoms (**Figure 1**). The coexistence of hematological disorders and auricular chondritis implied the existence of VEXAS syndrome. Re-evaluating the BM smear at the time of MM diagnosis revealed scattered granulocyte precursor cells containing cytoplasmic vacuoles (**Figure 2D**) in addition to the clonal expansion of plasma cells (**Figure 2D**). Genomic DNA from peripheral blood leukocytes was subjected to Sanger sequencing and peptide nucleic acid-clamping PCR of *UBA1*, identifying a somatic variant (NM_003334.3:c.122T>C:p.Met41Thr) (**Figure 3**) that strongly supported the diagnosis of VEXAS syndrome (1). After beginning the treatment for chondritis, PSL progressively reduced over a period of 7 months without any inflammatory reactions or progression of hematopoietic dysfunction.

## Discussion

VEXAS syndrome is a recently identified disease characterized by systemic inflammation and progressive BM cell dysplasia (10) caused by the somatic *UBA1* variants in hematopoietic cells (3). *UBA1* is an X-linked gene necessary for ubiquitination that plays a crucial role in the immune system; dysregulated ubiquitination can lead to systemic inflammatory responses (11). In the present case of coexisting auricular chondritis and MM, a somatic variant in *UBA1* (c.122T>C) resulted in amino acid substitution at codon 41 (p.Met41Thr), which has previously been reported in patients with VEXAS syndrome (1). Auricular chondritis, as observed in our case, is one of the most common clinical features of VEXAS syndrome.

A strong association exists between *UBA1* variants and the risk of MDS. However, MM on a background of VEXAS syndrome seems to be relatively rare, and it has not been elucidated whether *UBA1* is the driver gene for plasma cell dyscrasia such as MM (1, 5). It was also demonstrated that *UBA1* variants only occurred in hematopoietic cell populations that matured into myeloid cells, and were not seen in B or T lymphocytes in VEXAS syndrome (1). Obiorah et al. reported two cases of patients with VEXAS syndrome and MM with *UBA1* variant (p.Met41Leu) without MDS (5); however, the detailed treatment course or the origin of its somatic variant was unexplained in the report. In our case of VEXAS syndrome with a somatic *UBA1* variant (p.Met41Thr), the typical pathological findings of MM without myeloid dysplastic changes were demonstrated. Although somatic *UBA1* variants are known to cause chondritis, the relation of *UBA1* variants with MM remains elusive.

Previous reports have suggested that elderly patients with RP are at risk of MDS or hematological malignancy (12). Additionally, previous studies demonstrated the co-occurrence of RP or VEXAS syndrome and B-cell or plasma cell neoplasms, but these patients were not complicated with MDS (**Table 1**) (1, 5, 13–16). In a multicenter case series of 116 French patients with VEXAS syndrome, MDS was identified in 58/116 cases (50%), among which 12 cases were identified as having monoclonal gammopathy of undetermined significance (MGUS) (17). However, it is unclear whether somatic *UBA1* variants induce the development of MM or whether pathological conditions, such as MGUS or plasma cell disorders, could arise from somatic *UBA1* variants. Further investigations to uncover the detailed molecular mechanisms are needed.

**Table 1:** B-cell or plasma cell neoplasms in patients with auricular chondritis or VEXAS syndrome.

Case	Year	Number	Gender	Age of onset (years)	Diagnosis of RP	Diagnosis of VEXAS	Site of malignancy
Francès C, et al. (13)	2001	2	1 M/1 F	Mean age: 61	+	?	IgA myeloma
Sato K, et al. (14)	2006	1	M	51	+	?	Meningeal plasma cell granuloma
Castrejón I, et al. (15)	2007	1	M	67	+	?	Lymphoplasmocytic lymphoma
Hayashi S, et al. (16)	2017	1	M	77	+	?	Meningeal plasma cell granuloma
Beck DB, et al. (1)	2020	4	M	Mean age: 66	+	+	MM or MGUS
Obiorah IE, et al. (5)	2021	4	M	Mean age: 63	NA	+	MM (1/4), MGUS (1/4), MBL (1/4), MM+MBL (1/4)

VEXAS, Vacuoles, E1 enzyme; X-linked, autoinflammatory somatic syndrome; RP, relapsing polychondritis; M, male; F, female; Ig, immunoglobulin; MM, multiple myeloma; MGUS, monoclonal gammopathy of undetermined significance; NA, not applicable; MBL, Monoclonal B-cell lymphosis.

+, is diagnosed; ?, indicates that the disease concept of VEXAS syndrome is unknown since the disease concept of VEXAS syndrome was not known at that time, and NA indicates that the diagnosis of RP is not described.

In conclusion, we identified a somatic *UBA1* variant in a patient with auricular chondritis and MM. The relationship between VEXAS syndrome and plasma cell disorders, including MM, remains unclear. However, plasma cell disorders are rarely associated with patients with chondritis, and hence the somatic variants of *UBA1* should be screened in such patients.

## Data Availability Statement

The original contributions presented in the study are included in the article/**Supplementary Material**. Further inquiries can be directed to the corresponding author.

## Ethics Statement

The studies involving human participants were reviewed and approved by the Ethics Committee of Fukushima Medical University (2019-141). The patients/participants provided their written informed consent to participate in this study. Written informed consent was obtained from the individual(s) for the publication of any potentially identifiable images or data included in this article.

## Author Contributions

HM, TA, KM were involved with the conception of the work. YF, KY, SY, JT, NaokiM, MF, SS and HW contributed to the treatment and collection of data. MF, TI and NS performed histopathological evaluation of the bone marrow specimens. NT, AM, YU, YK and NaomichiM performed the experiments for genetic validation. MM, HS, CS, KK, performed colonoscopy. HM, YF, KM wrote the first draft of the manuscript. All authors contributed to the article and approved the submitted version.

## Funding

This study was supported by the JSPS KAKENHI (grant numbers JP20K17428 to NT, JP21k15097 to YU) and the Japan Agency for Medical Research and Development (AMED) under grant numbers JP21ek0109486, JP21ek0109549, JP21cm0106503, and JP21ek0109493 to NM. Additionally, this study was supported by the Japan Grant-in-Aid for Scientific Research (JP20K08777) to KM.

## Conflict of Interest

The authors declare that the research was conducted in the absence of any commercial or financial relationships that could be construed as a potential conflict of interest.

## Publisher’s Note

All claims expressed in this article are solely those of the authors and do not necessarily represent those of their affiliated organizations, or those of the publisher, the editors and the reviewers. Any product that may be evaluated in this article, or claim that may be made by its manufacturer, is not guaranteed or endorsed by the publisher.
